# Trends in Socioeconomic Inequalities in Cancer Screening Participation Before and After the COVID-19 Pandemic in Japan

**DOI:** 10.2188/jea.JE20250021

**Published:** 2025-10-05

**Authors:** Tshewang Gyeltshen, Hirokazu Tanaka, Kota Katanoda

**Affiliations:** 1 School of International Health, The University of Tokyo, Tokyo, Japan; 2 Division of Population Data Science, National Cancer Center Institute for Cancer Control, Tokyo, Japan

**Keywords:** socioeconomic inequalities, cancer screening, COVID-19, Japan

## Abstract

**Background:**

Socioeconomic inequalities in cancer screening participation remain a public health issue worldwide. We assessed trends in cancer screening participation according to socioeconomic status in Japan between 2013 and 2022, considering the potential impact of the coronavirus disease 2019 (COVID-19) pandemic.

**Methods:**

Data from the nationally representative Comprehensive Survey of Living Conditions (2013–2022: approximately 500,000 persons per survey) were analyzed for age-standardized self-reported cancer screening rates for stomach, lung, colon, breast (aged 40–69 years), and cervical (aged 20–69 years) cancers, stratified by education levels. An age-adjusted Poisson model was used to assess the statistical significance of changes between the survey years.

**Results:**

A clear socioeconomic gradient was observed, particularly in stomach cancer screening, where the 2022 rates ranged from 28.3% (low education) to 58.2% (high education) for men and 20.2% to 43.2% for women, depending on education level. Between 2019 and 2022, screening rates for stomach, lung, and colorectal cancers changed by −1.2%, −0.9%, and +0.6% for men and −1.0%, +0.1%, and +1.4% for women, respectively. Breast and cervical cancer screening rates declined by 0.5% and 0.4%, respectively. The COVID-19 pandemic worsened inequalities, with a 3.1% decline in breast cancer screening among individuals with low education level, compared to a 1.0% decline among those with higher education level.

**Conclusion:**

The COVID-19 pandemic had a minor impact on screening rates (counteracting increasing trends of screening rates), except for colorectal cancer screening rates; however, the impact was relatively severe for individuals with lower socioeconomic status, especially for women.

## INTRODUCTION

Cancer is a major public health burden worldwide. Over the years, increasing trends in cancer have strained health systems in both developing and developed countries. It is estimated that 28.4 million new cases of cancer and 16.3 million cancer-related deaths will be diagnosed by 2040.^1^ Enhancing the efficiency of cancer detection methods not only alleviates the organizational and individual burdens but also reduces mortality rates by enabling early detection.^2^ Organized screening initiatives for cancer and other non-communicable diseases have been shown to improve screening rates.^3^^,^^4^ While concerted screening efforts are made through various approaches, many people still miss out on the opportunity to undergo cancer screening.^5^ Additionally, socioeconomic differences in cancer screening may cause inequalities in cancer outcomes.^6^ The coronavirus disease 2019 (COVID-19) pandemic has exacerbated these issues, leading to changes in mortality rates, disruptions in health services, and alterations in medical care provision, particularly evident from 2020 to 2022.^7^ Previous studies have reported low participation rates in various cancer screenings among women with lower education levels and non-permanent workers in Japan.^8^^,^^9^

Since 1981, cancer has become the leading cause of death in Japan, surpassing cardiovascular diseases.^10^ The high cancer prevalence in Japan imposes a significant health burden and is only projected to increase.^11^ Over the past few decades, Japan has witnessed a rise in the number of cancer cases, primarily attributed to an aging population, lifestyle changes, and improved diagnostic capabilities.^12^ While the overall age-standardized mortality rate is decreasing, the incidence of screening-related cancers has not decreased.^13^

However, there has been no comprehensive report on the trends in cancer screening participation rates for both sexes, specifically stratified based on socioeconomic status. Moreover, while the COVID-19 pandemic affected cancer screening programs in Japan, the assessments of these impacts are lacking. Therefore, this study is the first to evaluate the effect of the COVID-19 pandemic on cancer screening trends in Japan, including the data collected after the COVID-19 pandemic in 2022.

## METHODS

### Study design, settings, and data sources

We analyzed data from the Comprehensive Survey of Living Conditions (CSLC) from 2013 to 2022. This large-scale national population-based survey has been conducted every 3 years since 1986 by the Ministry of Health, Labour and Welfare (MHLW) in Japan. The CSLC survey had major revisions of questionnaires after 2013. Therefore, we included only the survey data after 2013 for consistency. The CSLC has a sample size of approximately 500,000 people per survey. It collects comprehensive nationwide data on various aspects of living conditions, including family composition, occupation, income, and health.^14^ A component of the CSLC is the cancer screening questionnaire, which asks people if they have been screened for stomach, lung, colorectal, breast, and cervical cancers in the past 1–2 years. This includes participation in organized and opportunistic screening initiatives. The response rates for the survey were 79.6%, 77.6%, 72.5%, and 68.4% for the survey years 2013, 2016, 2019, and 2022, respectively. The percentage of missing responses for cancer screening rates was under 10% for each survey year and cancer type.

### Study variables

#### Definition of cancer screening

Self-reported cancer screening participation was assessed based on participants’ responses to the question, “Have you undergone screening for a specific cancer in the past 1–2 years?” Individuals who responded affirmatively (“Yes”) for each of the five cancer sites, stomach (past 1 year), lung (past 1 year), colorectal (past 1 year), breast (past 2 years), and cervical (past 2 years) cancers were considered to have undergone cancer screening for that cancer within the specified survey year. The analysis included participants aged 40–69 years for stomach, lung, colon, and breast cancer screening, while those aged 20–69 years were analyzed for cervical cancer screening.

#### Socioeconomic status

To assess socioeconomic status, we utilized educational level as a main indicator. Data on educational levels have been available since 2010; however, we used only data after 2013 owing to a major revision of the CSLC survey questionnaire after 2013, as discussed above. Educational level was categorized into the following three groups: “low” (corresponding to International Standard Classification of Education [ISCED] levels 1–2), “middle” (corresponding to ISCED levels 3–4), and “high” (corresponding to ISCED levels 5–8). In the Japanese education system, the “low” category represents individuals who have completed elementary school or junior high school, the “middle” category includes high school graduates and graduates of technical professional schools, and the “high” category includes individuals who have completed a 2-year college, university, or graduate school.^14^ We also considered occupational class as a sub-socioeconomic indicator because a cancer screening program is mainly provided in line with workplace health promotion in Japan. Based on occupational class, individuals were classified into the following five categories: upper non-manual workers (eg, professionals and managers), lower non-manual workers (eg, clerical, service, and sales workers), manual workers (eg, craft and related trades workers and semi-skilled and unskilled manual workers), farmers, and self-employed. This classification followed the Erikson-Goldthorpe-Portocarero scheme.^14^

#### Statistical analysis

We examined cancer screening rates for lung, stomach, and colorectal cancers for both men and women as well as for breast and cervical cancers for women only based on their education levels. The survey participants were presented in terms of frequencies and proportions with percentages. To account for demographic imbalances in the survey participants, all analyses were performed using weights derived from the weighting scores provided by the MHLW. A weighted analysis stratified based on educational level (2013–2022) was conducted to calculate cancer screening rates across Japan. Individuals with unknown education levels were excluded from the analysis for educational levels. A similar analysis was conducted based on occupational class, and all survey data and results are presented in eTable 1, eTable 2, eTable 3, eTable 4, eTable 5, and eTable 6.

Age-standardized cancer screening rates were calculated using the 2015 Japanese Standard Population.^15^ The absolute difference in screening rates between the successive survey years was calculated by subtracting the cancer screening rate of the previous survey year from that of the survey year that followed (eg, 2013–2016). Changes before (2016–2019) and after (2019–2022) the COVID-19 pandemic were reported in the same manner to assess the impact of the COVID-19 pandemic on cancer screening rates. In addition to calculating the absolute differences in screening rates, age-adjusted Poisson regression models were used to assess statistically significant changes between the survey years.

## RESULTS

### Characteristics of survey participants

The characteristics of the survey participants are shown in Table 1 (all survey data are presented in eTable 1). The population-weighted percentage of men (aged 40–69 years) with “high” and “middle” education levels changed from 36.3% and 50.9% in 2013 to 44.4% and 49.5% in 2022, respectively. Over the same survey period, the population-weighted percentages of women (aged 20–69 years) classified under “high” and “middle” education groups increased, starting at 37.6% and 55.3% in 2013 and ending at 46.3% and 50.0% in 2022, respectively.

**Table 1.** Number of survey participants (men aged 40–69 years and women aged 20–69 years)

Survey year	2013			2016			2019			2022		
	*n*	(%)	Weighted (%)	*n*	(%)	Weighted (%)	*n*	(%)	Weighted (%)	*n*	(%)	Weighted (%)
Men												
All population (aged 40–69 years)	122,431			118,817			110,667			95,553		
Educational level (aged 40–69 years)												
Low (ISCED: 1, 2)	12,981	12.0	12.8	9,622	9.5	10.2	7,209	7.7	8.8	5,284	6.5	6.2
Middle (ISCED: 3, 4)	59,662	55.2	50.9	56,115	55.3	51.5	51,771	55.0	51.0	43,846	53.7	49.5
High (ISCED: 5–8)	35,488	32.8	36.3	35,736	35.2	38.3	35,139	37.3	40.2	32,555	39.9	44.4
Unknown	14,300	—	—	17,344	—	—	16,548	—	—	13,868	—	—
Women												
All population (aged 40–69 years)	130,164			125,503			115,900			100,462		
Educational level (aged 40–69 years)												
Low (ISCED: 1, 2)	11,979	10.5	9.9	8,271	7.8	7.5	5,347	5.5	5.8	3,449	4.0	4.1
Middle (ISCED: 3, 4)	72,761	63.5	56.8	66,719	62.7	55.9	59,944	61.1	54.5	50,310	58.7	53.2
High (ISCED: 5–8)	29,775	26.0	33.3	31,478	29.6	36.6	32,756	33.4	39.7	32,005	37.3	42.7
Unknown	15,649	—	—	19,035	—	—	17,853	—	—	14,698	—	—
All population (aged 20–69 years)	192,761			174,313			162,201			138,670		
Educational level (aged 20–69 years)												
Low (ISCED: 1, 2)	14,200	8.3	7.2	9,741	6.5	5.7	6,724	4.9	5.4	4,401	3.7	3.7
Middle (ISCED: 3, 4)	102,160	59.6	55.3	88,677	59.1	54.5	79,829	57.7	52.2	66,038	55.2	50.0
High (ISCED: 5–8)	55,017	32.1	37.6	51,603	34.4	39.8	51,706	37.4	42.3	49,159	41.1	46.3
Unknown	21,384	—	—	25,456	—	—	23,942	—	—	19,072	—	—

ISCED, International Standard Classification of Education.

Low (ISCED: 1, 2): elementary school/junior high school graduation.

Middle (ISCED: 3, 4): high school/technical professional school graduation.

High (ISCED: 5–8): 2-year college/university graduation and more.

### Cancer screening rates

Table 2 and Table 3 show the screening rates for stomach, lung, and colorectal cancers among men and women, respectively. Table 4 shows the screening rates for breast and cervical cancer. The overall cancer screening rates as reported are shown in eFigure 1.

**Table 2.** Trends in age-standardized cancer screening rates by educational level for men (stomach, lung, and colorectal cancer: 40–69 years, past 1 year)

Survey year	2013		2016		2019		2022		Changes 2013–2016		Changes 2016–2019 (before COVID-19 pandemic)		Changes 2019–2022 (after COVID-19 pandemic)	
	%	95% CI	%	95% CI	%	95% CI	%	95% CI	Absolute difference (% point)^*^	*P* value	Absolute difference (% point)	*P* value	Absolute difference (% point)	*P* value
Stomach														
All population (40–69 years)	49.4	(49.1–49.7)	48.4	(48.1–48.6)	49.7	(49.4–50.0)	48.5	(48.2–48.8)	−1.1	<0.001	+1.4	<0.001	−1.2	<0.001
Educational level														
Low (ISCED: 1, 2)	28.5	(27.5–29.5)	28.4	(27.4–29.5)	28.7	(27.5–29.8)	28.3	(27.1–29.6)	−0.1	0.738	+0.2	0.737	−0.3	0.928
Middle (ISCED: 3, 4)	46.2	(45.8–46.7)	44.9	(44.5–45.4)	45.8	(45.4–46.3)	44.1	(43.6–44.5)	−1.3	0.002	+0.9	0.030	−1.8	<0.001
High (ISCED: 5–8)	59.6	(59.1–60.1)	58.8	(58.3–59.3)	59.4	(58.9–59.9)	58.2	(57.7–58.7)	−0.8	0.162	+0.6	0.234	−1.2	0.019
Lung														
All population (40–69 years)	51.8	(51.5–52.1)	53.3	(53.0–53.6)	55.4	(55.2–55.7)	54.5	(54.2–54.8)	+1.5	<0.001	+2.1	<0.001	−0.9	0.003
Educational level														
Low (ISCED: 1, 2)	33.5	(32.4–34.5)	36.8	(35.6–37.9)	37.9	(36.7–39.1)	35.8	(34.4–37.1)	+3.3	<0.001	+1.1	0.827	−2.1	0.125
Middle (ISCED: 3, 4)	49.8	(49.4–50.2)	51.2	(50.7–51.6)	53.1	(52.7–53.6)	51.9	(51.4–52.4)	+1.4	0.003	+2.0	<0.001	−1.2	0.007
High (ISCED: 5–8)	60.2	(59.7–60.7)	62.0	(61.6–62.5)	63.5	(63.1–64.0)	62.9	(62.4–63.4)	+1.8	0.002	+1.5	0.007	−0.6	0.183
Colorectal														
All population (40–69 years)	45.4	(45.1–45.7)	46.9	(46.6–47.2)	49.5	(49.2–49.8)	50.1	(49.8–50.4)	+1.5	<0.001	+2.6	<0.001	+0.6	0.052
Educational level														
Low (ISCED: 1, 2)	27.1	(26.1–28.0)	28.9	(27.8–30.0)	30.2	(29.0–31.4)	30.5	(29.3–31.8)	+1.9	0.041	+1.3	0.647	+0.3	0.809
Middle (ISCED: 3, 4)	42.6	(42.1–43.0)	44.1	(43.6–44.5)	46.5	(46.0–46.9)	46.8	(46.3–47.2)	+1.5	<0.001	+2.4	<0.001	+0.3	0.422
High (ISCED: 5–8)	54.7	(54.2–55.2)	56.4	(55.9–56.9)	58.6	(58.1–59.0)	58.9	(58.4–59.3)	+1.7	0.001	+2.1	<0.001	+0.3	0.606

CI, confidence interval; COVID-19, coronavirus disease 2019; ISCED, International Standard Classification of Education.

^*^Calculated from percentage of two time points.

Low (ISCED: 1, 2): elementary school/junior high school graduation.

Middle (ISCED: 3, 4): high school/technical professional school graduation.

High (ISCED: 5–8): 2-year college/university graduation and more.

**Table 3.** Trends in age-standardized cancer screening rates by educational level for women (stomach, lung, and colorectal cancer: 40–69 years, past 1 year)

Survey year	2013		2016		2019		2022		Changes 2013–2016		Changes 2016–2019 (before COVID-19 pandemic)		Changes 2019–2022 (after COVID-19 pandemic)	
	%	95% CI	%	95% CI	%	95% CI	%	95% CI	Absolute difference (% point)^*^	*P* value	Absolute difference (% point)^*^	*P* value	Absolute difference (% point)^*^	*P* value
Stomach														
All population (40–69 years)	37.0	(36.8–37.3)	37.0	(36.7–37.3)	38.2	(37.9–38.5)	37.2	(36.9–37.4)	0.0	0.828	+1.2	<0.001	−1.0	<0.001
Educational level														
Low (ISCED: 1, 2)	24.5	(23.4–25.6)	24.1	(22.8–25.3)	24.2	(22.9–25.6)	20.2	(18.8–21.6)	−0.5	0.089	+0.2	0.702	−4.0	<0.001
Middle (ISCED: 3, 4)	35.4	(35.0–35.8)	35.8	(35.4–36.2)	35.8	(35.4–36.2)	34.3	(33.9–34.7)	+0.4	0.278	0.0	0.947	−1.5	<0.001
High (ISCED: 5–8)	43.2	(42.5–43.8)	42.8	(42.3–43.4)	44.3	(43.8–44.8)	43.2	(42.7–43.7)	−0.3	0.840	+1.5	<0.001	−1.1	0.043
Lung														
All population (40–69 years)	41.2	(40.9–41.5)	43.5	(43.2–43.8)	47.1	(46.8–47.4)	47.2	(46.9–47.5)	+2.3	<0.001	+3.6	<0.001	0.1	0.765
Educational level														
Low (ISCED: 1, 2)	27.9	(26.8–29.1)	29.2	(27.8–30.5)	32.5	(31.0–34.0)	27.8	(26.3–29.4)	+1.2	0.028	+3.3	0.018	−4.7	<0.001
Middle (ISCED: 3, 4)	40.6	(40.2–41.0)	43.2	(42.8–43.6)	45.7	(45.3–46.1)	46.1	(45.7–46.5)	+2.6	<0.001	+2.4	<0.001	+0.4	0.436
High (ISCED: 5–8)	46.3	(45.7–46.9)	48.2	(47.7–48.8)	52.6	(52.1–53.1)	52.3	(51.8–52.8)	+2.0	<0.001	+4.4	<0.001	−0.4	0.552
Colorectal														
All population (40–69 years)	37.7	(37.4–38.0)	40.0	(39.7–40.3)	42.1	(41.8–42.3)	43.5	(43.2–43.7)	+2.3	<0.001	+2.1	<0.001	+1.4	<0.001
Educational level														
Low (ISCED: 1, 2)	25.1	(24.0–26.2)	25.9	(24.6–27.2)	27.1	(25.7–28.5)	23.3	(21.8–24.7)	+1.0	0.649	+1.2	0.408	−3.9	0.002
Middle (ISCED: 3, 4)	36.4	(36.0–36.8)	38.9	(38.5–39.3)	40.0	(39.6–40.4)	41.2	(40.7–41.6)	+2.5	<0.001	+1.1	0.003	+1.2	0.003
High (ISCED: 5–8)	43.9	(43.3–44.5)	46.4	(45.8–46.9)	48.6	(48.0–49.1)	49.7	(49.2–50.2)	+2.4	<0.001	+2.2	<0.001	+1.1	0.007

CI, confidence interval; COVID-19, coronavirus disease 2019; ISCED, International Standard Classification of Education.

^*^Calculated from percentage of two time points.

Low (ISCED: 1, 2): elementary school/junior high school graduation.

Middle (ISCED: 3, 4): high school/technical professional school graduation.

High (ISCED: 5–8): 2-year college/university graduation and more.

**Table 4.** Trends in age-standardized cancer screening rates by educational level for women (breast and cervical cancer: past 2 year)

Survey year	2013		2016		2019		2022		Changes 2013–2016		Changes 2016–2019 (before COVID-19 pandemic)		Changes 2019–2022 (after COVID-19 pandemic)	
	%	95% CI	%	95% CI	%	95% CI	%	95% CI	Absolute difference (% point)^*^	*P* value	Absolute difference (% point)^*^	*P* value	Absolute difference (% point)^*^	*P* value
Breast														
All population (40–69 years)	43.5	(43.2–43.8)	46.5	(46.2–46.7)	48.7	(48.4–49.0)	48.2	(47.9–48.5)	+3.0	<0.001	+2.2	<0.001	−0.5	0.073
Educational level														
Low (ISCED: 1, 2)	26.8	(25.7–27.9)	28.4	(27.0–29.7)	28.4	(27.0–29.8)	25.3	(23.8–26.8)	+1.5	0.053	0.0	0.457	−3.1	0.001
Middle (ISCED: 3, 4)	41.8	(41.4–42.2)	44.7	(44.3–45.1)	45.7	(45.3–46.1)	44.7	(44.3–45.1)	+2.9	<0.001	+1.0	0.010	−1.0	0.012
High (ISCED: 5–8)	52.9	(52.3–53.5)	54.3	(53.7–54.8)	57.2	(56.7–57.7)	56.2	(55.7–56.7)	+1.4	0.011	+2.9	<0.001	−1.0	0.057
Cervical														
All population (20–69 years)	41.6	(41.4–41.8)	43.2	(42.9–43.4)	44.2	(44.0–44.4)	43.8	(43.6–44.0)	+1.6	<0.001	+1.0	<0.001	−0.4	0.069
Educational level														
Low (ISCED: 1, 2)	29.3	(28.3–30.3)	29.6	(28.4–30.8)	29.4	(28.1–30.6)	28.6	(27.2–30.0)	+0.4	0.431	−0.2	0.804	−0.8	0.063
Middle (ISCED: 3, 4)	39.8	(39.5–40.1)	41.7	(41.3–42.0)	41.6	(41.2–41.9)	40.7	(40.4–41.1)	+1.9	<0.001	−0.1	0.657	−0.8	0.007
High (ISCED: 5–8)	48.3	(47.9–48.8)	48.6	(48.2–49.1)	50.4	(50.0–50.8)	49.2	(48.8–49.6)	+0.3	0.808	+1.7	<0.001	−1.2	0.002

CI, confidence interval; COVID-19, coronavirus disease 2019; ISCED, International Standard Classification of Education.

^*^Calculated from percentage of two time points.

Low (ISCED: 1, 2): elementary school/junior high school graduation.

Middle (ISCED: 3, 4): high school/technical professional school graduation.

High (ISCED: 5–8): 2-year college/university graduation and more.

### Screening for stomach cancer

Overall, stomach cancer screening has been relatively stable over the years. In 2022, stomach cancer screening rates for low, middle, and high education levels were 28.3%, 44.1%, and 58.2% for men and 20.2%, 34.3%, and 43.2% for women, respectively. Among men, those with high educational levels had the highest screening rates across all surveyed years, starting at 59.6% in 2013 and ending at 58.2% in 2022. In contrast, men with low educational levels had the lowest screening rates, starting from 28.5% in 2013 to 28.3% in 2022. The middle educational group experienced a decline in screening rates, from 46.2% in 2013 to 44.1% in 2022. Similar patterns were observed among women (Table 3). The screening rates for stomach cancer increased significantly between the 2013–2016 and 2016–2019 survey periods, with changes of −1.1% and +1.4% among men, and 0.0% and +1.2% among women, respectively (*P* < 0.001). However, a significant decline in screening rates was observed between 2019–2022, with reductions of −1.2% for men and −1.0% for women (*P* < 0.001). The decline was particularly pronounced among men with a middle level of education (−1.8%; *P* < 0.001) and women with a low level of education (−4.0%; *P* < 0.001).

When stratified based on occupational class, upper non-manual workers showed the highest screening rates throughout the survey period, whereas self-employed individuals and economically inactive individuals showed the lowest screening rates. The changes before and after the pandemic vary in each occupational class; however, after the pandemic, the screening rates declined among upper non-manual workers, self-employed individuals, and economically inactive individuals. eTable 2 presents the detailed results of occupational class.

### Screening for lung cancer

The overall screening rate for lung cancer increased between the baseline year (2013: 51.8% for men and 41.2% for women) and the final year (2022: 54.5% for men and 47.2% for women) among individuals aged 40–69 years (Table 2 and Table 3). In 2022, lung cancer screening rates for low, middle, and high education levels were 35.8%, 51.9%, and 62.9% for men and 27.8%, 46.1%, and 52.3% for women, respectively. The lung cancer screening rates increased significantly between the 2013–2016 and 2016–2019 survey with the net absolute change of 1.5% and 2.1% respectively for men and 2.3% and 3.6% respectively for women (*P* < 0.001). However, the screening rates for lung cancer during the COVID-19 pandemic (2016–2022) slightly declined among men (−0.9%; *P* = 0.003) and remained unchanged for women (0.1%; *P* = 0.765). The decrease between 2019 and 2022 was more pronounced among those with lower educational levels for both men (−2.1%) and women (−4.7%) than among those with higher educational levels (men: −0.6%; women: −0.4%).

Upper non-manual workers showed the highest screening rates throughout the survey period, whereas self-employed individuals and economically inactive individuals showed the lowest screening rates. After the pandemic, the screening rates declined among upper non-manual workers, self-employed individuals, and economically inactive individuals. eTable 3 presents the detailed results of occupational class.

### Screening for colorectal cancer

Overall, colorectal cancer screening rates increased substantially from 2013 to 2022 in both men (45.4% to 50.1%) and women (37.7% to 43.5%), as shown in Table 2 and Table 3. In 2022, colorectal cancer screening rates for low, middle, and high education levels were 30.5%, 46.8%, and 58.9% for men and 23.3%, 41.2%, and 49.7% for women, respectively. The screening rates for colorectal cancer (all population) between 2013–2016 and 2016–2019 significantly increased for men (+1.5% and +2.6% respectively; *P* < 0.001) and women (+2.3% and +2.1% respectively; *P* < 0.001). Notably, colorectal cancer screening rates increased during the COVID-19 pandemic, with a rise of +0.6% among men (*P* = 0.052) and +1.4% among women (*P* < 0.001) in 2022. However, between 2019 and 2022, a decline was observed among those with lower educational levels for women (−3.9%; *P* = 0.002).

Similar to findings of the screening for lung and stomach cancers, colorectal cancer demonstrated the highest screening rates among the upper non-manual workers throughout the survey period and the lowest screening rates among self-employed and economically inactive individuals. Detailed results of occupational class are provided in eTable 4.

### Screening for breast cancer

Breast cancer screening rates for women aged 40–69 years increased by 4.7% from 2013 (43.5%) to 2022 (48.2%). In 2022, breast cancer screening rates for low, middle, and high education levels were 25.3%, 44.7%, and 56.2% for women, respectively (Table 4). When stratified based on occupational class, upper non-manual workers had the highest breast cancer screening rates (eTable 5). The screening rates for breast cancer (all population) between 2013–2016 and 2016–2019 significantly increased (+3.0% and +2.2% respectively; *P* < 0.001). However, during the COVID-19 pandemic (2019–2022), the screening rate for breast cancer (all population) slightly declined, with a 0.5% decrease in absolute difference. This decline was most noticeable in the low education group, which experienced the highest absolute change among the three educational groups (−3.1%; *P* = 0.001).

### Screening for cervical cancer

The cervical cancer screening rate among women aged 20–69 years (Table 4) increased significantly by 2.2%, from 41.6% in 2013 to 43.8% in 2022. In 2022, cervical cancer screening rates for low, middle, and high education levels were 28.6%, 40.7%, and 49.2% for women, respectively. The screening rates for cervical cancer (all population) between 2013–2016 and 2016–2019 significantly increased (+1.6% and +1.0% respectively; *P* < 0.001). Following the COVID-19 pandemic (2019–2022), the screening rate change for cervical cancer (all population) remained approximately the with absolute difference of −0.4% (*P* = 0.069). When stratified based on occupational class, upper non-manual workers had the highest cervical cancer screening rates (eTable 6).

## DISCUSSION

This study identified the existence of significant inequalities in cancer screening rates across different socioeconomic statuses in Japan. Following the COVID-19 pandemic, we observed a slight decline in screening rates, except for that of colorectal cancer, which could be attributed to the effects of the pandemic. The cancer screening rates were observed to be higher among individuals with higher education levels, leading to widening socioeconomic inequalities in cancer screening, regardless of the COVID-19 pandemic.

Overall, we observed increased cancer screening rates across different cancer types, with a slight decrease in the post-pandemic years. While secular trends increased over the years, substantial differences exist between sexes and different socioeconomic strata. Individuals in the lower socioeconomic status groups and women consistently had lower screening rates over the survey years. Individuals with higher and middle educational levels exhibited higher screening rates than those with lower educational levels. Among men, lung cancer screening had the highest rate across the years, reaching 54.5% in 2022. Colorectal cancer screening followed closely at 50.1%, and stomach cancer screening at 48.5%. Similarly, women had higher screening rates for lung cancer than for other cancer types. Across all socioeconomic strata and cancer sites, the screening rate has been shown to be affected by the COVID-19 pandemic.

Our findings indicated that, although there has been a consistent increase in cancer screening rates in the general population, considerable variations in the screening rates exist across different education levels, occupational classes, genders, and cancer types. In both sexes, the percentage of individuals during the survey years indicated a shift toward higher levels of education, with a concurrent decrease in the proportion of women with low education levels. Although cancer screening rates gradually increased across socioeconomic groups, they remained low among individuals with lower education levels.

We observed that the cancer screening rates were highly unequal across different socioeconomic strata. People in the middle and higher socioeconomic strata (middle and high educational levels) had higher screening uptake rates than those in the lower socioeconomic strata. These findings have been reported in previous studies across different population subgroups in Japan.^16^^,^^17^ Consistent with our findings, similar screening inequalities have been reported internationally across East Asia.^18^^,^^19^ These socioeconomic inequalities are not limited to cancer and affect other health outcomes in Japan (eg, educational inequalities in mortality^20^).

When analyzing the screening uptake rates based on cancer types, all cancers assessed in this study showed increasing uptake rates until 2019. Screening rates for sex-inclusive cancers (stomach, lung, and colon) were higher among men than among women. In both sexes across all types of cancer, individuals in the upper occupational and educational strata showed higher annual uptake changes than those in the lower strata. All cancer types exhibited increased secular trends over the survey years. This is likely owing to the robust cancer control policies implemented in Japan over the years through the programmatic policy approaches since the creation of the National Cancer Center in 1962.^21^^,^^22^

Our findings are consistent with those of previous studies. For instance, consistent with our results, an earlier study reported higher screening rates among Japanese men.^17^ Japan is generally perceived as a patriarchal society, where men typically hold defining roles in employment and household provision.^23^^,^^24^ The introduction of comprehensive preventive, diagnosis, and screening policies over the years,^25^ with focused screening initiatives in communities and workplaces, may have attributed to this gender disparity.^26^ Another study on socioeconomic inequalities in cancer screening among women revealed a widening gap in participation rates across different educational levels, which are similar to our results.^8^

We noticed a decline in cancer screening rates in 2022 across all cancer types, except for colorectal cancer. Consistent upward trends were found in screening uptake over the years until 2019, but 2022 stands out. We attributed this phenomenon to the impact of the COVID-19 pandemic, which started in 2020 and continued through early 2023 in Japan. The impact of COVID-19 has been evident across many health outcomes apart from cancer screening.^27^ This effect was globally felt as the pandemic burdened healthcare systems, restricting movements and accessibility.^28^^,^^29^ Consistent with these studies, our findings indicate the impact of COVID-19 on cancer screening uptake rates in Japan. The higher absolute decrease among individuals in the lower educational level groups suggests widening socioeconomic inequalities in cancer screening in the country. Earlier studies that utilized different data sources showed similar impacts of COVID-19 on cancer screening across various cancer types in Japan.^30^^,^^31^ Further, a recent study on the impact of COVID-19 on breast cancer screening using the same dataset reported a decline in breast cancer screening rates among the municipality-based screening while worksite-based screening is reported to have increased.^32^ However, declines in cancer screening rates among male upper non-manual workers should be noted carefully (shown in eTable 2, eTable 3, and eTable 4). The pandemic has also been shown to impact cancer diagnosis in Japan, particularly during the early days of the pandemic.^33^^,^^34^ A similar decline in cancer screening during the COVID-19 pandemic was also reported in neighboring South Korea.^35^

Our findings highlight the need for targeted interventions to bridge this inequity gap and ensure equitable access to cancer prevention services in Japan. To address these socioeconomic inequalities, targeted interventions, such as awareness and outreach, health education, and access to screenings aimed at individuals of lower education and socioeconomic strata, should be implemented. Previous research has indicated a positive impact of using free screening coupons on cancer screening uptake. A study analyzing the CSLC data shows that providing free coupon increases the probability of taking screening by 10% and 9% respectively for breast and cervical cancer screenings.^36^ In Japan, providing free coupons has been discontinued in 2013 transitioning to local governance. The effect of such program was lauded to be heterogenous; while the overall screening uptake increased, benefits were disproportionately accrued by people of higher socioeconomic status, aligning with the inverse equity hypothesis.^37^^,^^38^ For example, Tabuchi et al reports that reduction of out-of-pocket costs through distribution of vouchers has increased cancer screening uptake rates among females and proven to have reduced inequalities in breast cancer screening while at the same time widening the inequalities in cervical cancer screening participation. The United States Community Preventive Services Task Force currently recommends reducing out-of-pocket costs for breast cancer screening, citing insufficient evidence for similar strategies in other cancer screening.^39^ We believe that financial incentives alone may not be sufficient to ensure equitable screening uptake and that targeted, multi-faceted interventions are required to reduce cancer screening inequalities effectively. Eliminating this inequity will ensure equitable access to nationwide cancer screening services across Japan.

### Limitations

The CSLC utilizes a robust methodology, ensuring representative sampling for generalizability. The data represent self-reported cancer screening participation within the past 1–2 years prior to the time of individual’s response. This survey offers a valuable alternative source for estimating national screening rates in settings, such as Japan, where data from different health centers are not easily integrated. The overall trends observed in this study closely correspond with the published reports from the MHLW. Nevertheless, it is important to acknowledge some limitations. First, the reliance on self-reported data introduces the possibility of recall bias,^40^ potentially impacting the accuracy of the study findings. Second, educational level alone may not reflect the true socioeconomic status of the individuals, as socioeconomic variables can comprise multiple defining variables. Nonetheless, occupational stratification has been provided in the appendix for reference. Thirdly, the CSLC includes lung cancer screening using simple chest X-rays, a modality that is not widely recommended in international practice. However, this approach remains endorsed in the most recent Japanese Lung Cancer Screening Guidelines (2006 edition) and continues to be part of current national practice.^41^^,^^42^ It is important to acknowledge that international comparisons involving our lung cancer screening data may be influenced by this difference between Japanese screening practices and international best practice recommendations.

### Conclusions

Cancer screening participation rates increased overall in Japan from 2013 to 2022; however, socioeconomic inequities were substantial. Individuals with higher socioeconomic status (higher education and occupational class) exhibited consistently higher cancer screening rates over the survey years, whereas those with lower socioeconomic status, such as individuals with low education levels and manual workers, consistently reported lower cancer screening rates. The COVID-19 pandemic led to a slight decline in screening rates (counteracting increasing trends of screening rates), except for colorectal cancer screening in 2022, contributing to widening socioeconomic inequalities in Japan. Promotional programs for cancer screening are necessary, particularly for lower socioeconomic groups.

## SUPPLEMENTARY MATERIAL

eTable 1. Number of survey participants

eTable 2. Trends in age-standardized cancer screening rates by occupational class and educational level (stomach, 40–69 years)

eTable 3. Trends in age-standardized cancer screening rates by occupational class and educational level (lung, 40–69 years)

eTable 4. Trends in age-standardized cancer screening rates by occupational class and educational level (colorectal, 40–69 years)

eTable 5. Trends in age-standardized cancer screening rates by occupational class and educational level (breast, 40–69 years)

eTable 6. Trends in age-standardized cancer screening rates by occupational class and educational level (cervical, 20–69 years)

eFigure 1. Age-standardized self-reported cancer screening rate for men and women from 2013–2022, Japan
