# Supplementary material for: Trends in Socioeconomic Inequalities in Cancer Screening Participation Before and After the COVID-19 Pandemic in Japan
Source: J Epidemiol. 2025 Oct 5;35(10):451–9. doi: 10.2188/jea.JE20250021 (PMC12420948; doi:10.2188/jea.JE20250021)
Supplement: Supplementary file 1 [file je-35-451-s001.pdf]

eTable 1. Number of survey participants

Survey year	2013			2016			2019			2022		
	n	(%)	Weighted	n	(%)	Weighted	n	(%)	Weighted	n	(%)	Weighted
Men												
All population (aged 40–69 years)	122,431			118,817			110,667			95,553		
Occupational class (EGP scheme, aged 40–69 years)												
Upper non-manual workers (I+II)	30,788	34.8	30.1	30,360	34.9	30.2	30,144	36.0	31.4	26,062	36.0	37.1
Lower non-manual workers (III)	20,066	22.7	24.0	19,661	22.6	24.1	18,590	22.2	24.1	16,599	22.9	24.8
Manual workers (V+VI+VIIa)	20,392	23.1	23.2	21,076	24.2	24.4	21,125	25.2	25.1	18,941	26.1	24.2
Farmers (IVc+VIIb)	4,916	5.6	4.4	4,624	5.3	4.2	4,073	4.9	4.0	3,163	4.4	2.8
Self-employed (IVa+b)	12,278	13.9	18.3	11,375	13.1	17.1	9,784	11.7	15.5	7,717	10.6	11.2
Economically inactive/unknown	32,038		-	29,859		-	25,230		-	18,111	-	-
Educational level (aged 40–69 years)												
Low (ISCED: 1, 2)	12,981	12.0	12.8	9,622	9.5	10.2	7,209	7.7	8.8	5,284	6.5	6.2
Middle (ISCED: 3, 4)	59,662	55.2	50.9	56,115	55.3	51.5	51,771	55.0	51.0	43,846	53.7	49.5
High (ISCED: 5–8)	35,488	32.8	36.3	35,736	35.2	38.3	35,139	37.3	40.2	32,555	39.9	44.4
Unknown	14,300			17,344			16,548			13,868		
Women												
All population (aged 20–69 years)	192,761			174,313			162,201			138,670		
Occupational class (EGP scheme, aged 20–69 years)												
Upper non-manual workers (I+II)	27,961	25.3	24.5	26,790	25.7	25.0	27,879	27.3	25.0	25,516	28.4	27.8
Lower non-manual workers (III)	62,225	56.3	58.5	58,358	56.0	58.1	55,982	54.9	56.5	48,952	54.5	56.6
Manual workers (V+VI+VIIa)	12,309	11.1	10.6	11,768	11.3	10.8	11,593	11.4	11.5	9,920	11.0	10.3
Farmers (IVc+VIIb)	3,463	3.1	2.0	3,206	3.1	2.0	2,828	2.8	2.3	2,179	2.4	1.6
Self-employed (IVa+b)	4,522	4.1	4.3	4,109	3.9	4.1	3,668	3.6	4.7	3,269	3.6	3.7
Economically inactive/unknown	77,391		-	65,078		-	54,665		-	43,641	-	-
Educational level (aged 20–69 years)												
Low (ISCED: 1, 2)	14,200	8.3	7.2	9,741	6.5	5.7	6,724	4.9	5.4	4,401	3.7	3.7
Middle (ISCED: 3, 4)	102,160	59.6	55.3	88,677	59.1	54.5	79,829	57.7	52.2	66,038	55.2	50.0
High (ISCED: 5–8)	55,017	32.1	37.6	51,603	34.4	39.8	51,706	37.4	42.3	49,159	41.1	46.3
Unknown	21,384		-	25,456		-	23,942		-	19,072	-	-

EGP scheme, Erikson-Goldthorpe-Portocarero scheme; ISCED, International Standard Classification of Education.

Low (ISCED: 1, 2): elementary school/junior high school graduation

Middle (ISCED: 3, 4): high school/technical professional school graduation

High (ISCED: 5–8): 2-year college/university graduation and more

eTable 2. Trends in age-standardized cancer screening rates by occupational class and educational level (stomach, 40–69 years)

Survey year	2013		2016		2019		2022		Changes 2013–2016	Changes 2016–2019	Changes 2019–2022
	%	95% CI	%	95% CI	%	%	%	95% CI	%	%	%
Men											
All population (40–69 years)	49.4	(49.1 – 49.7)	48.4	(48.1 – 48.6)	49.7	(49.4 – 50.0)	48.5	(48.2 – 48.8)	–1.1*	+1.4*	-1.2*
Occupational class (EGP scheme)											
Upper non-manual workers	65.4	(64.8 – 66.0)	63.7	(63.1 – 64.3)	65.5	(64.9 – 66.0)	63.2	(62.6 – 63.8)	-1.7*	+1.8*	-2.3*
Lower non-manual workers	55.5	(54.7 – 56.3)	54.7	(54.0 – 55.4)	55.6	(54.8 – 56.3)	55.9	(55.2 – 56.6)	-0.8	+0.8	+0.4
Manual workers	42.6	(41.8 – 43.4)	41.7	(41.0 – 42.4)	43.9	(43.2 – 44.6)	44.3	(43.6 – 45.1)	-0.9	+2.2*	+0.5
Farmers	41.8	(39.9 – 43.8)	40.9	(39.0 – 42.7)	39.2	(37.3 – 41.2)	39.3	(37.3 – 41.3)	-1.0	-1.6	+0.1
Self-employed	29.5	(28.7 – 30.4)	28.9	(28.1 – 29.8)	29.3	(28.4 – 30.1)	27.7	(26.8 – 28.6)	-0.6	+0.3	-1.6
Economically inactive/unknown	34.8	(34.1 – 35.5)	33.1	(32.4 – 33.8)	32.7	(32.0 – 33.4)	29.9	(29.2 – 30.6)	-1.7*	-0.4	-2.8*
Educational level (40–69 years)											
Low (ISCED: 1, 2)	28.5	(27.5 – 29.5)	28.4	(27.4 – 29.5)	28.7	(27.5 – 29.8)	28.3	(27.1 – 29.6)	-0.1	+0.2	-0.3
Middle (ISCED: 3, 4)	46.2	(45.8 – 46.7)	44.9	(44.5 – 45.4)	45.8	(45.4 – 46.3)	44.1	(43.6 – 44.5)	-1.3*	+0.9*	-1.8*
High (ISCED: 5–8)	59.6	(59.1 – 60.1)	58.8	(58.3 – 59.3)	59.4	(58.9 – 59.9)	58.2	(57.7 – 58.7)	-0.8	+0.6	-1.2*
Women											
All population (40–69 years)	37.0	(36.8 – 37.3)	37.0	(36.7 – 37.3)	38.2	(37.9 – 38.5)	37.2	(36.9 – 37.4)	0.0	+1.2*	-1.0*
Occupational class (EGP scheme)											
Upper non-manual workers	47.9	(46.9 – 48.9)	49.0	(48.1 – 49.9)	48.7	(47.9 – 49.5)	48.4	(47.6 – 49.2)	1.1	-0.3	-0.3
Lower non-manual workers	39.1	(38.6 – 39.7)	39.8	(39.3 – 40.4)	41.7	(41.2 – 42.2)	41.6	(41.0 – 42.1)	0.7	+1.9*	-0.2
Manual workers	35.7	(34.6 – 36.8)	33.5	(32.4 – 34.5)	34.3	(33.3 – 35.4)	33.9	(32.8 – 34.9)	-2.2*	+0.9	-0.5
Farmers	40.2	(37.7 – 42.7)	38.1	(35.8 – 40.3)	35.1	(32.8 – 37.5)	35.3	(32.8 – 37.8)	-2.1	-2.9*	+0.2
Self-employed	29.6	(28.1 – 31.2)	30.4	(28.9 – 32.0)	31.5	(29.9 – 33.1)	27.3	(25.7 – 28.9)	0.8	+1.0	-4.2*
Economically inactive/unknown	31.4	(31.0 – 31.9)	30.7	(30.3 – 31.1)	30.7	(30.3 – 31.2)	34.8	(34.3 – 35.3)	-0.7*	0.0	+4.1*
Educational level (40–69 years)											
Low (ISCED: 1, 2)	24.5	(23.4 – 25.6)	24.1	(22.8 – 25.3)	24.2	(22.9 – 25.6)	20.2	(18.8 – 21.6)	-0.5	+0.2	-4.0*
Middle (ISCED: 3, 4)	35.4	(35.0 – 35.8)	35.8	(35.4 – 36.2)	35.8	(35.4 – 36.2)	34.3	(33.9 – 34.7)	+0.4	0.0	-1.5*
High (ISCED: 5–8)	43.2	(42.5 – 43.8)	42.8	(42.3 – 43.4)	44.3	(43.8 – 44.8)	43.2	(42.7 – 43.7)	-0.3	+1.5*	-1.1*

CI, confidence interval; EGP scheme, Erikson-Goldthorpe-Portocarero scheme; ISCED, International Standard Classification of Education.

*P value <0.05

Low (ISCED: 1, 2): elementary school/junior high school graduation

Middle (ISCED: 3, 4): high school/technical professional school graduation

High (ISCED: 5–8): 2-year college/university graduation and more

eTable 3. Trends in age-standardized cancer screening rates by occupational class and educational level (lung, 40–69 years)

Survey year	2013		2016		2019		2022		Changes 2013–2016	Changes 2016–2019	Changes 2019–2022
	%	95% CI	%	95% CI	%	95% CI	%	95% CI	%	%	%
Men											
All population (40–69 years)	51.8	(51.5 – 52.1)	53.3	(53.0 – 53.6)	55.4	(55.2 – 55.7)	54.5	(54.2 – 54.8)	+1.5*	+2.1*	-0.9*
Occupational class (EGP scheme)											
Upper non-manual workers	66.3	(65.7 – 66.9)	67.7	(67.2 – 68.3)	69.5	(69.0 – 70.0)	68.1	(67.5 – 68.6)	+1.4	+1.7*	-1.4*
Lower non-manual workers	57.0	(56.2 – 57.8)	58.3	(57.6 – 59.1)	60.1	(59.4 – 60.8)	60.3	(59.6 – 61.0)	+1.4	+1.8*	+0.2
Manual workers	50.0	(49.2 – 50.8)	51.4	(50.6 – 52.1)	54.0	(53.3 – 54.7)	54.4	(53.6 – 55.1)	+1.4	+2.7*	+0.3
Farmers	44.4	(42.4 – 46.3)	46.6	(44.7 – 48.5)	45.9	(43.9 – 47.9)	45.5	(43.4 – 47.5)	+2.2	-0.7	-0.5
Self-employed	33.4	(32.5 – 34.2)	34.8	(33.9 – 35.7)	36.2	(35.3 – 37.2)	34.4	(33.4 – 35.4)	+1.4	+1.5	-1.8
Economically inactive/unknown	35.5	(34.7 – 36.2)	36.1	(35.4 – 36.8)	36.8	(36.0 – 37.5)	34.4	(33.7 – 35.1)	+0.6	+0.7	-2.4*
Educational level (40–69 years)											
Low (ISCED: 1, 2)	33.5	(32.4 – 34.5)	36.8	(35.6 – 37.9)	37.9	(36.7 – 39.1)	35.8	(34.4 – 37.1)	+3.3*	+1.1	-2.1
Middle (ISCED: 3, 4)	49.8	(49.4 – 50.2)	51.2	(50.7 – 51.6)	53.1	(52.7 – 53.6)	51.9	(51.4 – 52.4)	+1.4*	+2.0*	-1.2*
High (ISCED: 5–8)	60.2	(59.7 – 60.7)	62.0	(61.6 – 62.5)	63.5	(63.1 – 64.0)	62.9	(62.4 – 63.4)	+1.8*	+1.5*	-0.6
Women											
All population (40–69 years)	41.2	(40.9 – 41.5)	43.5	(43.2 – 43.8)	47.1	(46.8 – 47.4)	47.2	(46.9 – 47.5)	+2.3*	+3.6*	+0.1
Occupational class (EGP scheme)											
Upper non-manual workers	54.7	(53.8 – 55.7)	59.3	(58.4 – 60.1)	62.1	(61.4 – 62.9)	62.3	(61.6 – 63.1)	+4.5*	+2.9*	+0.2
Lower non-manual workers	44.3	(43.7 – 44.9)	46.4	(45.9 – 47.0)	51.5	(51.0 – 52.1)	52.4	(51.9 – 52.9)	+2.2*	+5.1*	+0.9
Manual workers	42.6	(41.5 – 43.8)	42.9	(41.8 – 44.0)	46.3	(45.2 – 47.4)	48.3	(47.2 – 49.4)	+0.2	+3.4*	+2.0
Farmers	44.5	(42.0 – 47.1)	46.4	(44.1 – 48.7)	44.8	(42.4 – 47.3)	43.6	(41.0 – 46.2)	+1.9	-1.6	-1.2
Self-employed	30.6	(29.0 – 32.2)	33.9	(32.4 – 35.5)	38.0	(36.3 – 39.6)	34.5	(32.8 – 36.2)	+3.3*	+4.0*	-3.5*
Economically inactive/unknown	33.4	(33.0 – 33.9)	34.7	(34.2 – 35.1)	36.3	(35.9 – 36.8)	34.8	(34.3 – 35.3)	+1.3*	+1.7*	-1.5*
Educational level (40–69 years)											
Low (ISCED: 1, 2)	27.9	(26.8 – 29.1)	29.2	(27.8 – 30.5)	32.5	(31.0 – 34.0)	27.8	(26.3 – 29.4)	+1.2*	+3.3*	-4.7*
Middle (ISCED: 3, 4)	40.6	(40.2 – 41.0)	43.2	(42.8 – 43.6)	45.7	(45.3 – 46.1)	46.1	(45.7 – 46.5)	+2.6*	+2.4*	+0.4
High (ISCED: 5–8)	46.3	(45.7 – 46.9)	48.2	(47.7 – 48.8)	52.6	(52.1 – 53.1)	52.3	(51.8 – 52.8)	+2.0*	+4.4*	-0.4

CI, confidence interval; EGP scheme, Erikson-Goldthorpe-Portocarero scheme; ISCED, International Standard Classification of Education.
*P value <0.05

Low (ISCED: 1, 2): elementary school/junior high school graduation

Middle (ISCED: 3, 4): high school/technical professional school graduation

High (ISCED: 5–8): 2-year college/university graduation and more

eTable 4. Trends in age-standardized cancer screening rates by occupational class and educational level (colorectal, 40–69 years)

Survey year	2013		2016		2019		2022		Changes 2013–2016	Changes 2016–2019	Changes 2019–2022
	%	95% CI	%	95% CI	%	95% CI	%	95% CI	%	%	%
Men											
All population (40–69 years)	45.4	(45.1 – 45.7)	46.9	(46.6 – 47.2)	49.5	(49.2 – 49.8)	50.1	(49.8 – 50.4)	+1.5*	+2.6*	+0.6
Occupational class (EGP scheme)											
Upper non-manual workers	58.6	(58.0 – 59.2)	59.8	(59.2 – 60.4)	63.1	(62.5 – 63.6)	62.8	(62.2 – 63.4)	+1.1*	+3.3*	-0.3
Lower non-manual workers	50.2	(49.4 – 51.0)	51.7	(51.0 – 52.4)	54.3	(53.6 – 55.0)	56.7	(55.9 – 57.4)	+1.4	+2.6*	+2.3*
Manual workers	39.9	(39.2 – 40.7)	42.0	(41.2 – 42.7)	45.3	(44.6 – 46.0)	47.7	(47.0 – 48.5)	+2.0*	+3.3*	+2.5*
Farmers	39.4	(37.5 – 41.3)	40.5	(38.6 – 42.4)	41.4	(39.4 – 43.3)	44.4	(42.4 – 46.3)	+1.1	+0.9	+3.0
Self-employed	30.5	(29.7 – 31.4)	31.8	(30.9 – 32.7)	33.1	(32.2 – 34.0)	35.0	(34.0 – 35.9)	+1.3	+1.3	+1.8
Economically inactive/unknown	32.2	(31.5 – 32.9)	32.5	(31.8 – 33.1)	32.8	(32.1 – 33.6)	33.6	(33.0 – 34.3)	+0.3	+0.4	+0.8
Educational level (40–69 years)											
Low (ISCED: 1, 2)	27.1	(26.1 – 28.0)	28.9	(27.8 – 30.0)	30.2	(29.0 – 31.4)	30.5	(29.3 – 31.8)	+1.9*	+1.3	+0.3
Middle (ISCED: 3, 4)	42.6	(42.1 – 43.0)	44.1	(43.6 – 44.5)	46.5	(46.0 – 46.9)	46.8	(46.3 – 47.2)	+1.5*	+2.4*	+0.3
High (ISCED: 5–8)	54.7	(54.2 – 55.2)	56.4	(55.9 – 56.9)	58.6	(58.1 – 59.0)	58.9	(58.4 – 59.3)	+1.7*	+2.1*	+0.3
Women											
All population (40–69 years)	37.7	(37.4 – 38.0)	40.0	(39.7 – 40.3)	42.1	(41.8 – 42.3)	43.5	(43.2 – 43.7)	+2.3*	+2.1*	+1.4*
Occupational class (EGP scheme)											
Upper non-manual workers	46.0	(45.0 – 47.0)	49.4	(48.5 – 50.3)	51.5	(50.7 – 52.3)	53.3	(52.5 – 54.2)	+3.4*	+2.1*	+1.8*
Lower non-manual workers	39.4	(38.8 – 40.0)	41.9	(41.4 – 42.5)	45.5	(44.9 – 46.0)	47.3	(46.7 – 47.8)	+2.5*	+3.5*	+1.8*
Manual workers	36.5	(35.4 – 37.6)	37.7	(36.7 – 38.8)	40.7	(39.6 – 41.7)	42.9	(41.8 – 44.0)	+1.2	+2.9*	+2.2*
Farmers	40.6	(38.1 – 43.1)	40.8	(38.5 – 43.0)	37.9	(35.5 – 40.3)	42.1	(39.7 – 44.5)	0.0	-2.8	+4.2
Self-employed	32.0	(30.4 – 33.6)	34.3	(32.8 – 35.9)	36.4	(34.8 – 38.1)	35.3	(33.6 – 36.9)	+2.3*	+2.1	-1.2
Economically inactive/unknown	33.3	(32.8 – 33.7)	34.6	(34.2 – 35.1)	34.4	(33.9 – 34.9)	35.1	(34.7 – 35.6)	+1.4*	-0.3	+0.7
Educational level (40–69 years)											
Low (ISCED: 1, 2)	25.1	(24.0 – 26.2)	25.9	(24.6 – 27.2)	27.1	(25.7 – 28.5)	23.3	(21.8 – 24.7)	+1.0	+1.2	-3.9*
Middle (ISCED: 3, 4)	36.4	(36.0 – 36.8)	38.9	(38.5 – 39.3)	40.0	(39.6 – 40.4)	41.2	(40.7 – 41.6)	+2.5*	+1.1*	+1.2*
High (ISCED: 5–8)	43.9	(43.3 – 44.5)	46.4	(45.8 – 46.9)	48.6	(48.0 – 49.1)	49.7	(49.2 – 50.2)	+2.4*	+2.2*	+1.1*

CI, confidence interval; EGP scheme, Erikson-Goldthorpe-Portocarero scheme; ISCED, International Standard Classification of Education.

*P value <0.05

Low (ISCED: 1, 2): elementary school/junior high school graduation

Middle (ISCED: 3, 4): high school/technical professional school graduation

High (ISCED: 5–8): 2-year college/university graduation and more

eTable 5. Trends in age-standardized cancer screening rates by occupational class and educational level (breast, 40–69 years)

Survey year	2013		2016		2019		2022		Changes 2013–2016	Changes 2016–2019	Changes 2019–2022
	%	95% CI	%	95% CI	%	95% CI	%	95% CI	%	%	%
Women											
All population (40–69 years)	43.5	(43.2 – 43.8)	46.5	(46.2 – 46.7)	48.7	(48.4 – 49.0)	48.2	(47.9 – 48.5)	+3.0*	+2.2*	-0.5
Occupational class (EGP scheme)											
Upper non-manual workers	51.7	(50.8 – 52.6)	54.7	(53.8 – 55.5)	56.1	(55.3 – 56.9)	58.3	(57.6 – 59.1)	+2.9*	+1.4	+2.3
Lower non-manual workers	45.0	(44.5 – 45.6)	48.2	(47.7 – 48.8)	51.2	(50.7 – 51.7)	51.2	(50.7 – 51.7)	+3.2*	+3.0*	0.0
Manual workers	38.2	(37.1 – 39.2)	40.0	(38.9 – 41.1)	42.2	(41.1 – 43.3)	41.6	(40.5 – 42.7)	+1.8	+2.2*	-0.6
Farmers	45.0	(42.4 – 47.5)	48.5	(46.1 – 50.9)	51.2	(48.6 – 53.7)	46.8	(44.2 – 49.5)	+3.5*	+2.7	-4.4
Self-employed	38.2	(36.6 – 39.7)	40.4	(38.7 – 42.0)	44.6	(42.9 – 46.4)	41.6	(39.8 – 43.3)	+2.2*	+4.3*	-3.1
Economically inactive/unknown	39.7	(39.3 – 40.2)	42.2	(41.8 – 42.7)	43.4	(42.9 – 43.9)	41.3	(40.8 – 41.8)	+2.5*	+1.1*	-2.1*
Educational level (40–69 years)											
Low (ISCED: 1, 2)	26.8	(25.7 – 27.9)	28.4	(27.0 – 29.7)	28.4	(27.0 – 29.8)	25.3	(23.8 – 26.8)	+1.5	0.0	-3.1*
Middle (ISCED: 3, 4)	41.8	(41.4 – 42.2)	44.7	(44.3 – 45.1)	45.7	(45.3 – 46.1)	44.7	(44.3 – 45.1)	+2.9*	+1.0*	-1.0*
High (ISCED: 5–8)	52.9	(52.3 – 53.5)	54.3	(53.7 – 54.8)	57.2	(56.7 – 57.7)	56.2	(55.7 – 56.7)	+1.4*	+2.9*	-1.0

CI, confidence interval; EGP scheme, Erikson-Goldthorpe-Portocarero scheme; ISCED, International Standard Classification of Education.

*P value <0.05

Low (ISCED: 1, 2): elementary school/junior high school graduation

Middle (ISCED: 3, 4): high school/technical professional school graduation

High (ISCED: 5–8): 2-year college/university graduation and more

eTable 6. Trends in age-standardized cancer screening rates by occupational class and educational level (cervical, 20–69 years)

Survey year	2013				2016				2019				2022				Changes 2013–2016	Changes 2016–2019	Changes 2019–2022
	%	95% CI			%	95% CI			%	95% CI			%	95% CI			%	%	%
Women																			
All population (20–69 years)	41.6	(41.4	–	41.8)	43.2	(42.9	–	43.4)	44.2	(44.0	–	44.4)	43.8	(43.6	–	44.0)	+1.6*	+1.0*	-0.4
Occupational class (EGP scheme)																			
Upper non–manual workers	48.1	(47.4	–	48.8)	50.0	(49.3	–	50.6)	51.0	(50.4	–	51.6)	51.8	(51.2	–	52.4)	+1.9*	+1.1	+0.8
Lower non–manual workers	41.9	(41.5	–	42.3)	43.5	(43.1	–	43.9)	45.2	(44.8	–	45.6)	45.2	(44.8	–	45.6)	+1.6*	+1.7*	0.0
Manual workers	35.8	(34.9	–	36.8)	36.4	(35.5	–	37.3)	36.8	(35.8	–	37.7)	38.3	(37.3	–	39.3)	+0.6	+0.4	+1.5
Farmers	39.9	(37.4	–	42.5)	45.0	(42.0	–	47.9)	45.6	(42.9	–	48.4)	43.3	(40.5	–	46.1)	+5.4*	+0.7	-2.3
Self–employed	37.5	(35.8	–	39.2)	39.4	(37.2	–	41.6)	41.6	(39.5	–	43.8)	37.9	(36.2	–	39.7)	+2.4*	+2.2	-3.7
Economically inactive/unknown	39.8	(39.4	–	40.1)	41.1	(40.7	–	41.5)	41.1	(40.7	–	41.5)	39.3	(38.8	–	39.8)	+1.4*	+0.0	-1.8*
Educational level (20–69 years)																			
Low (ISCED: 1, 2)	29.3	(28.3	–	30.3)	29.6	(28.4	–	30.8)	29.4	(28.1	–	30.6)	28.6	(27.2	–	30.0)	+0.4	-0.2	-0.8
Middle (ISCED: 3, 4)	39.8	(39.5	–	40.1)	41.7	(41.3	–	42.0)	41.6	(41.2	–	41.9)	40.7	(40.4	–	41.1)	+1.9*	-0.1	-0.8*
High (ISCED: 5–8)	48.3	(47.9	–	48.8)	48.6	(48.2	–	49.1)	50.4	(50.0	–	50.8)	49.2	(48.8	–	49.6)	+0.3	+1.7*	-1.2*

CI, confidence interval; EGP scheme, Erikson-Goldthorpe-Portocarero scheme; ISCED, International Standard Classification of Education.

*P value <0.05

Low (ISCED: 1, 2): elementary school/junior high school graduation

Middle (ISCED: 3, 4): high school/technical professional school graduation

High (ISCED: 5–8): 2-year college/university graduation and more

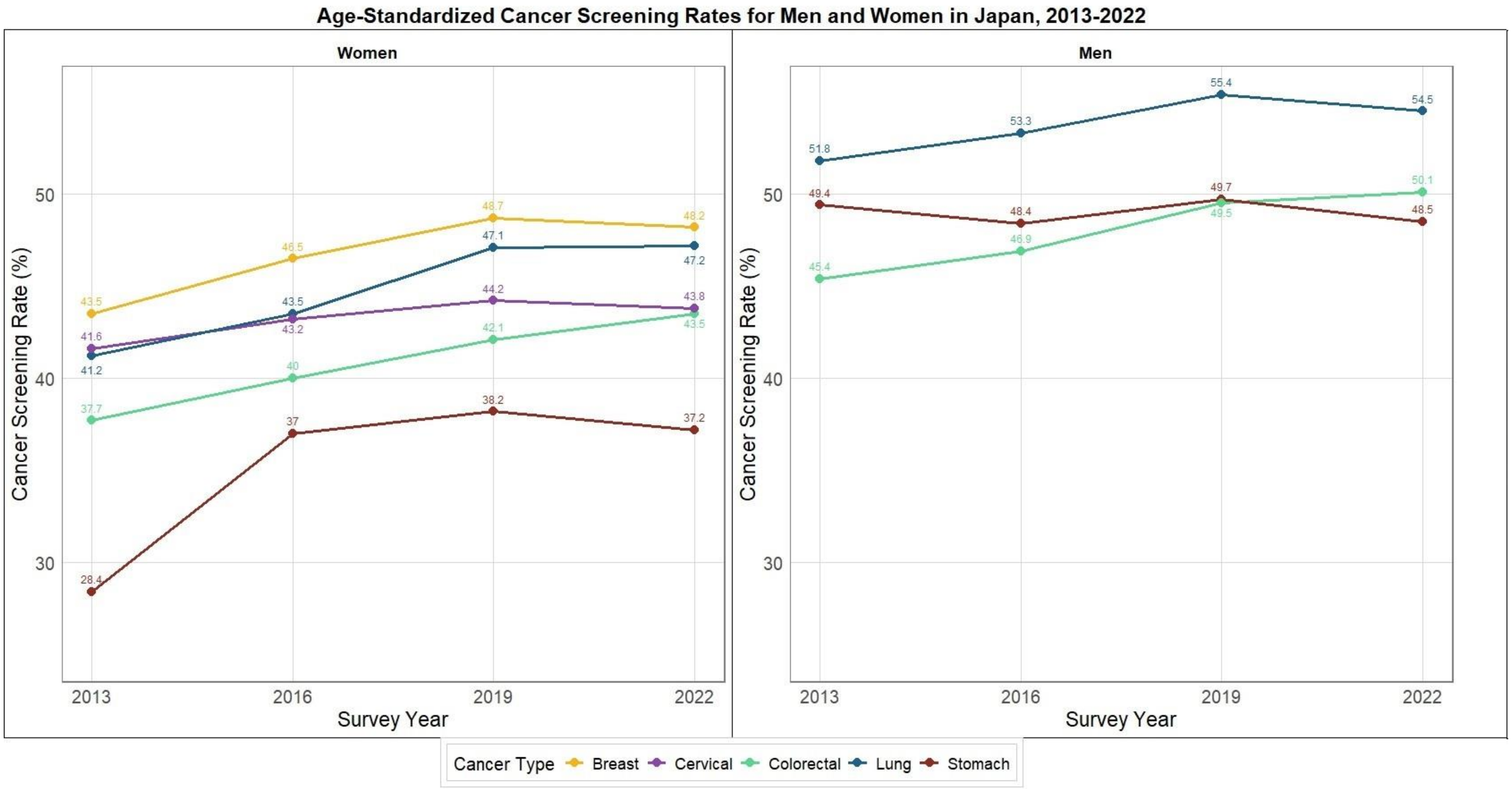

eFigure 1. Age-standardized self-reported cancer screening rate for men and women from 2013–2022, Japan
